# Was It Spontaneous Combustion?

**Published:** 1873-09

**Authors:** 


### WAS IT SPONTANEOUS COMBUSTION?

They report a queer case of spontaneous combustion from New Hampshire. A physician had prescribed linseed oil and camphor for a severe pain in the chest, and the patient complained of the heat soon after its application on cotton batting. In about an hour he protested he could bear it no longer, and before it could be removed, it took fire, actually blazing up and burning the poor fellow's neck severely.—*Med. and Surg. Reporter.*

